# MalariaNet: A Microcontroller-Deployable Malaria-Microscopy Detector for Point-of-Care Biosensing Under Leakage-Free Evaluation

**DOI:** 10.3390/bios16070358

**Published:** 2026-06-28

**Authors:** Mengdi Hou, Gaoming He, Zongchang Liu, Jianbo Huang, Heliang Zou

**Affiliations:** 1 Guangxi Key Laboratory of Machine Vision and Intelligent Control, Wuzhou University, Wuzhou 543000, China; houmengdi@gxuwz.edu.cn (M.H.); liuzongchang@gxuwz.edu.cn (Z.L.);; 2 School of Computer Application, Guilin University of Technology, Guilin 541004, China

**Keywords:** data leakage, slide-disjoint evaluation, reproducible benchmarking, malaria detection, embedded biosensing, lightweight CNN, point-of-care diagnostics, TinyML, microcontroller deployment, cross-site robustness

## Abstract

Compact malaria detectors for microcontrollers are almost always benchmarked on the NIH Malaria dataset with a per-cell random split. This leaks slide identity because the cells come from only about 200 slides and a random split mixes same-slide cells across training and testing. The leakage also distorts architectural conclusions: under a leakage-free slide-disjoint protocol, per-module ablation gains collapse to seed noise and an apparent cross-site robustness variant loses most of its advantage. Headline accuracy falls from 97.1% to 95.6%, a gap that sits within the cross-seed noise, and all eight tested architectures move the same way. The evidence is this unanimous direction, not the size of any single gap. This benchmarking finding is our main contribution. Two results survive. First, MalariaNet, our 21 K-parameter detector, reaches about 95.6% accuracy at 23.5 KB of INT8 weights, with a numerically faithful on-chip forward on an STM32H743 at a 1.2 FPS triage rate. Second, it is among the most interference-robust of the eight networks and the most robust microcontroller-deployable model. Scope is limited to single *P. falciparum* thin-smear cells. Slide-disjoint evaluation should become standard, and we provide MalariaNet as the first leakage-free, on-device-validated point-of-care malaria reference.

## 1. Introduction

Malaria ranks among the deadliest infectious diseases worldwide, with 249 million cases and 608,000 deaths in 2022, predominantly in sub-Saharan Africa [1]. The gold standard is microscopic examination of Giemsa-stained blood smears. It depends on trained microscopists, who are critically scarce in endemic regions. Each examination takes 20–30 min and exhibits significant inter-observer variability [2].

Deep learning has demonstrated strong potential for automated malaria detection [3,4]. However, nearly all existing systems depend on GPU servers; cloud connectivity; or, at minimum, smartphone-class hardware. This makes deployment in resource-limited environments challenging. The emerging paradigm of IoT-based edge intelligence runs AI inference directly on low-power microcontrollers (MCUs). This opens a new path toward point-of-care diagnostic tools that can operate without network connectivity or centralized infrastructure [5].

Modern MCUs such as the STM32H7 series feature a Cortex-M7 core up to 480 MHz, 2 MB Flash, and 1 MB RAM and cost under $10. They can serve as intelligent edge nodes in healthcare IoT ecosystems. Such nodes could, in principle, perform local inference, display results on-device, and optionally report aggregated findings to regional health authorities. To bring malaria detection onto these devices, however, the deployed models must be orders of magnitude smaller than conventional architectures while still preserving useful diagnostic accuracy.

Progress on automated malaria microscopy is measured almost entirely on one public benchmark, the NIH Malaria Cell Images dataset of 27,558 single-cell images. Crucially, these cells are not independent. They were segmented from the thin blood smears of roughly 200 patient slides, and the originating slide is encoded in every file name. Yet essentially all prior work, as well as the first version of this study, evaluates with a per-cell random split. Such a split places cells from the same slide into training, validation and test sets simultaneously. Cells from one slide share a staining batch, illumination, focus and background. Therefore, per-cell splitting leaks slide identity and optimistically biases every reported number. To the best of our knowledge, it is unknown whether the headline accuracies and architectural conclusions in the MCU malaria literature are inflated by slide leakage. This is the central concern of this paper.

To examine it under controlled conditions, we use a compact, interpretable, domain-aware detector, MalariaNet, as the testbed. Its design follows a two-domain decomposition of Giemsa microscopy. The first domain is a low-dimensional stain-colour cue in which parasites stain purple or blue and host cells stain pink or red. The second is multi-scale parasite morphology across life-cycle stages. This decomposition is realised by three-parameter frugal modules with 21 K parameters in total: a Stain-Colour Feature Extractor (SCFE) based on raw RGB, a Multi-Scale Morphology Encoder (MSME) with biologically motivated receptive fields, and a Cross-Domain Diagnostic Gate (CDDG) that fuses the two streams. We stress that we present this decomposition as an interpretable, MCU-frugal design, not as a set of empirically superior modules. As our results show, the apparent per-module advantages are, themselves, a slide-leakage artefact.

Our contributions, reported without selection, are the following:

- Primary contribution: Slide leakage inflates accuracy and distorts architectural conclusions on the NIH malaria benchmark. We re-evaluate an identical architecture under a leakage-free, slide-disjoint protocol: a group split on the slide identifier with three seeds. Accuracy then drops from 97.06±0.45% to 95.61±1.02%. This 1.45 pp fall is, itself, within the ±1.02 seed standard deviation. Therefore, our evidence is not the headline magnitude but the unanimous direction. Every one of the eight tested architectures inflated the same way, and every design conclusion below collapses. First, every per-module ablation gain reported under per-cell splitting—namely, +0.78/+0.38/+0.56/+0.37 pp—collapses to within seed noise and partly inverts, as detailed in Section 4.4. Second, a prototype-cosine classifier variant appears to deliver a striking +7.0 pp cross-site robustness gain under per-cell training. Retraining both models under the leakage-free protocol results in only +1.8 pp within ±5.5 pp seed noise. The “robustness variant” is, itself, a leakage artefact, as shown in Section 4.8. Thus, slide leakage invents plausible-looking innovations, not merely larger numbers. We recommend that slide-disjoint evaluation become standard for this benchmark.
- A leakage-free, on-device-validated compact baseline. Rigorous evaluation leaves a single validated artefact. Under the slide-disjoint protocol, the 21 K-parameter detector reaches ≈95.6% while fitting the STM32H743 envelope. On chip, it uses 23.5 KB INT8 weights and runs a measured 816 ms per 128×128 cell and a 1.23 FPS triage rate for a numerically faithful forward pass. INT8 preserves FP32 accuracy within 0.05 pp. This is a rigorously benchmarked, deployable reference for the cell-classification stage of point-of-care malaria microscopy. We validate it on device for accuracy, latency and memory; image acquisition, cell detection, slide-level aggregation and the wider IoT system are out of scope and are not discussed here.
- An interference-robustness advantage under controlled perturbations. The portable-microscope scenario actually requires resilience to imaging degradation. On this property, under a leakage-free, three-seed, all-CNN degradation sweep, the compact model is the second most robust of eight CNNs, with an accuracy drop of 21.8 pp from S0 to S5, as reported in Section 4.10. It trails only the 530× larger, non-deployable ResNet-18 and is, by a seed-consistent margin, the most robust MCU-deployable model. Unlike the per-module ablations and the prototype variant, this advantage survives leakage-free evaluation. Knowledge distillation from the leakage-free EfficientNet-B0 teacher additionally recovers ≈1/3 of the clean-accuracy gap, from 95.6% to 96.0%, at zero inference cost, as reported in Section 4.11. This is a partial mitigation, not a gap closer.

The remainder of this paper is organized as follows. Section 2 reviews MCU malaria detectors and the evaluation practices they adopt. Section 3 describes the MalariaNet testbed and the MalariaNet-P variant. Section 4 defines the per-cell vs. slide-disjoint protocols in Section 4.1.2 and reports leakage-free main results, the ablation collapse, on-device deployment, and cross-site transfer. Section 5 discusses the leakage finding and its implications for the field. Section 6 concludes this paper.

## 2. Related Work

### 2.1. Deep Learning for Malaria Detection on the NIH Benchmark

Deep learning has achieved high reported accuracy in automated malaria microscopy, overwhelmingly on the NIH Malaria Cell dataset. Mujahid et al. [2] reported high accuracy across benchmarks. Poostchi et al. [3] reviewed image analysis and machine learning for the task. Yang et al. [6] detected parasites in thick smears via intensity-based screening, followed by a customized CNN that reached 93.46% accuracy. Fuhad et al. [4] reported a 99.23%-accuracy smartphone-deployable system. Islam et al. [7] presented an explainable ViT, and Chaudhry et al. [8] proposed a <400 K-parameter architecture. Two properties are near-universal in this body of work and are central to our study. First, none targets MCU-class hardware. Second, all evaluate with a per-cell random split of the NIH cells, without controlling for the fact that those cells are segmented from a small number of patient slides. The accuracies reported above are, accordingly, per-cell figures; our work shows what they become under leakage-free evaluation.

### 2.2. Lightweight CNN Architectures

Efficient CNN design has been a major research focus. MobileNetV2 [9] introduced depthwise-separable convolutions and inverted residuals. ShuffleNetV2 [10] employed channel shuffle operations for efficient inter-group information exchange. EfficientNet [11] used NAS for optimal compound scaling. For MCU deployment, MCUNet [12] combined NAS with system-level optimization, achieving 70.7% ImageNet accuracy within 256 KB SRAM. These architectures represent the state of the art in generic efficient design, but they are task-agnostic. The same templates are applied regardless of the application domain.

### 2.3. TinyML and IoT in Healthcare

TinyML enables machine learning to run on ultra-low-power MCUs. It is supported by a mature ecosystem that includes TensorFlow Lite Micro [5], CMSIS-NN [13], and STM32Cube.AI. Within IoT-based digital healthcare, edge AI devices are envisioned as nodes that perform local inference without cloud dependency.

Ponnada et al. [14] demonstrated TinyML for health monitoring using optimized transfer learning, and Zeynali et al. [15] developed a TinyML-based blood glucose monitoring system on MCUs. However, TinyML applications in medical imaging remain largely underexplored, owing to the gap between the demands of image classification and the resource constraints of MCUs.

### 2.4. Data Leakage and Evaluation Rigour in Medical-Imaging ML

The reproducibility of machine learning-based science is increasingly recognised as fragile. Data leakage means information from the test set is inadvertently available at training time. It is identified as a pervasive cause of inflated, non-reproducible results across scientific ML [16]. In medical imaging, the canonical instance is subject-level leakage. Multiple images, such as slices, patches, or cells, often originate from the same patient or slide. A naive per-image random split then places correlated images from one subject into both the training and test sets, so the model is partly evaluated on subjects it has seen. The accepted remedy is grouped patient- or slide-wise splitting.

The NIH Malaria Cell dataset is a textbook case. Its 27,558 cells were segmented from ≈200 patient slides, and the slide identifier is embedded in every filename, yet the de facto protocol is a per-cell random split that leaks slides. This protocol was used in the works referenced in Section 2 and in the first version of this study. Stain normalisation and colour deconvolution [17] address a different source of inter-laboratory variability and do not mitigate this split-level leakage. To the best of our knowledge, no prior work has quantified how much slide leakage inflates results on this widely used, MCU-relevant benchmark or whether it changes architectural conclusions. No prior work has provided a leakage-free, on-device-validated baseline either. That gap is what this paper addresses.

## 3. Proposed Method

This section details MalariaNet, the compact two-stream detector used as the controlled testbed for this study. We stress at the outset what Section 4 establishes empirically. The stain–morphology decomposition described below is presented as an interpretable, MCU-frugal design, not as a set of empirically superior modules. Under leakage-free evaluation, the apparent per-module advantages are a slide-leakage artefact. Therefore, the module descriptions that follow motivate design choices. They should not be read as claims of validated per-module gains. We present the overall two-stream architecture, the three modules (SCFE, MSME, and CDDG), and the multi-task head and close with a per-module complexity analysis.

### 3.1. Overview

**Figure 1:**
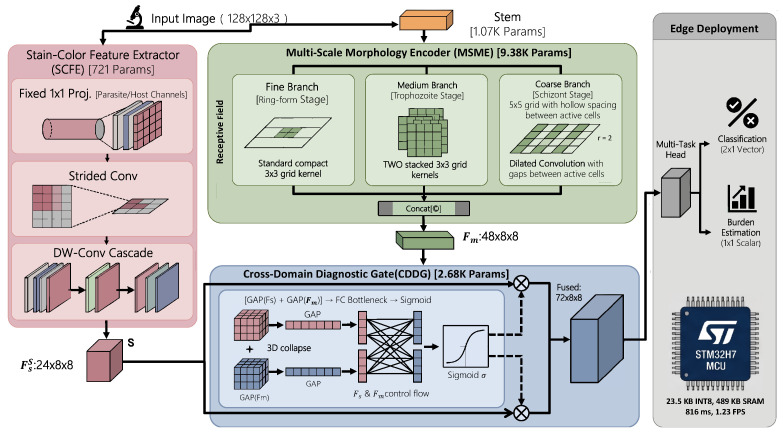
Architecture of MalariaNet. Two parallel streams (SCFE and MSME) are fused via CDDG for multi-task output.

Figure 1 illustrates the overall architecture of MalariaNet. Given a 128×128×3 input image, two parallel, domain-specific streams process it independently. SCFE operates directly on the raw RGB input to capture chromatic staining patterns. In parallel, a lightweight stem extracts low-level features at 32×32 resolution, which MSME then processes for structural features. The two streams are fused via CDDG, which adaptively weights each stream’s contribution. A multi-task head produces both infection classification and burden estimation outputs.

### 3.2. Morphology Stem

The stem preprocesses the input for the morphology stream. It consists of a 3×3 convolution (stride 2), a 3×3 depthwise convolution (stride 2), and a 1×1 pointwise convolution, each followed by batch normalization and ReLU6 activation. Together, these layers reduce spatial resolution from 128×128 to 32×32, expand the number of channels from 3 to 24, and consume only 1072 parameters in total.

The SCFE stream bypasses the stem entirely and operates directly on raw RGB. This deliberate asymmetry reflects the fundamentally different natures of the two signal domains.

### 3.3. Stain-Colour Feature Extractor (SCFE)

#### 3.3.1. SCFE Motivation

Giemsa staining produces a highly structured colour distribution in malaria blood-smear images. Parasitic structures absorb the basic dye component, staining purple/blue, while host red blood cell haemoglobin absorbs the acidic component, staining pink/red. This chromatic separation provides a fast discriminative cue that human microscopists exploit as a first-pass filter, and encoding it with a fixed projection rather than generic learned filters saves parameters. We treat this as a design hypothesis, not an established advantage. Under leakage-free evaluation, the stain stream provides no measurable in-domain accuracy gain, and the variant without it is, in fact, more accurate off-domain (Section 4.4 and Section 4.7). Therefore, SCFE is justified as a parameter-frugal, interpretable inductive bias rather than a validated biological prior.

#### 3.3.2. SCFE Design

Unlike the morphology stream, which operates on stem features, SCFE takes the raw RGB input image directly. It begins with a fixed, non-learnable 1×1 convolution that projects the 3-channel RGB to a 3-channel Giemsa stain-relevant representation. The projection matrix is expressed as follows:

(1) $$P=\left[\begin{array}{ccc} 0.15 & -0.70 & 0.70\\ 0.70 & 0.15 & -0.70\\ 0.58 & 0.58 & 0.58 \end{array}\right]$$

Row 1 encodes the purple/blue parasite response (high B, low R). Row 2 encodes the pink/red host-cell response (high R, low B). Row 3 encodes the luminance channel. These vectors are derived from Giemsa stain-colour deconvolution principles [18] and frozen during training, acting as a strong inductive bias that regularizes the colour stream.

**Figure 2:**
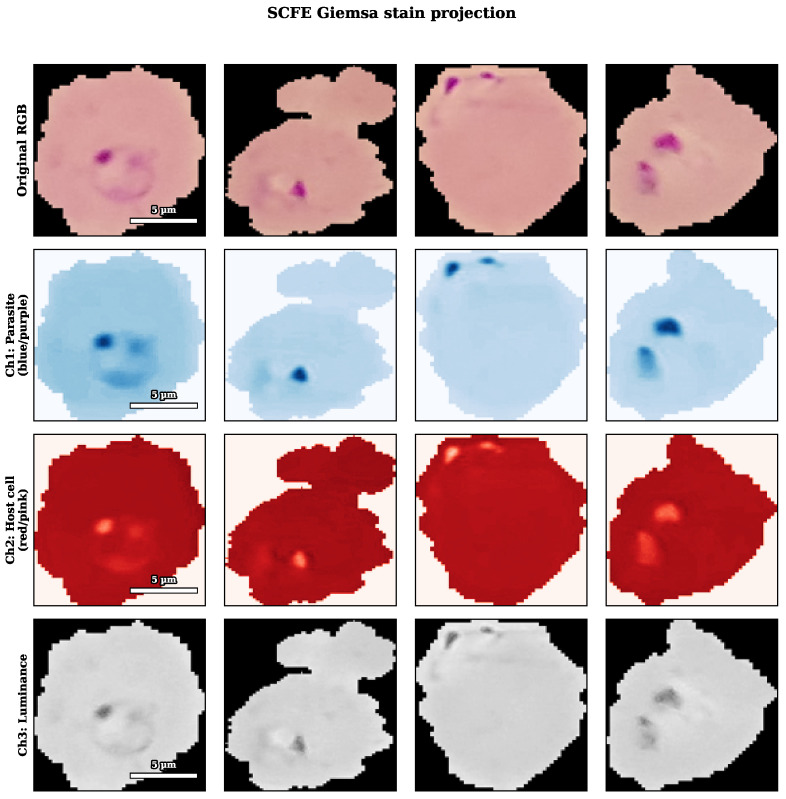
SCFE Giemsa stain projection. Row 1: original. Row 2: parasite channel. Row 3: host cell channel. Row 4: luminance. Each column is one cell shown across the four channels, so the four rows of a column share the same scale. As in Figure 3, the white bar in the first column of each row is an approximate ∼5 μm reference, and the absolute scale varies between columns because cells are individually segmented and resized to 128×128. Channels are shown in false colour, with the parasite channel in blue and the host-cell channel in red.

Following the fixed projection, a lightweight downsampling convolution (3×3, stride 4) reduces from 128×128 to 32×32. Two depthwise-separable convolution blocks then extract stain intensity patterns while reducing spatial resolution to 8×8. The entire SCFE uses approximately 721 learnable parameters, reflecting the inherently low-dimensional nature of colour information. It yields an output of $F_{s}\in R^{C_{s}\times 8\times 8}$ with $C_{s}=24$. Figure 2 visualizes the projection effect: the parasite channel highlights infected regions, while the host cell channel captures the red blood cell background.

### 3.4. Multi-Scale Morphology Encoder (MSME)

#### 3.4.1. MSME Motivation

Malaria parasites at different life-cycle stages present drastically different spatial scales in microscopy images:

- Ring-form trophozoites: 1–2 μm diameter, mapping to ∼2–4 pixels at 128×128 resolution and characterized by small, sharp chromatin dots;
- Mature trophozoites: 5–8 μm, mapping to ∼8–12 pixels, with a larger cytoplasmic area with visible pigment granules;
- Schizonts: 10–20 μm, mapping to ∼15–30 pixels and containing multiple merozoites with distinct boundaries.

A single receptive field cannot efficiently capture all scales. Standard multi-scale approaches such as FPN (∼1.2 M parameters in the lateral pyramid alone) and ASPP (∼0.5 M parameters for the dilated branches) are two to three orders of magnitude beyond the 21 K MCU budget targeted here.

#### 3.4.2. MSME Design

The MSME consists of two stages, each containing three parallel depthwise convolution branches with biologically motivated receptive fields:

- Fine branch: Standard 3×3 depthwise convolution, targeting ring-form structures and chromatin dots (2–5 pixel features);
- Medium branch: Two stacked 3×3 depthwise convolutions (effective 5×5 receptive field), targeting mature trophozoites (8–12 pixel features), with stacking avoiding the parameter cost of a direct 5×5 kernel;
- Coarse branch: 3×3 depthwise convolution with a dilation rate of 2, targeting schizonts and larger structures (15–30 pixel features). The medium and coarse branches share a 5×5 theoretical receptive field but differ critically in sampling pattern. The medium branch samples densely to capture continuous textures such as pigment granules. The coarse branch instead samples sparsely with gaps, capturing boundary and edge features at a wider spatial extent. After the stem’s 4× downsampling, the dilated receptive field covers ∼20 pixels in the original image, matching schizont dimensions.

The three branch outputs are concatenated and fused with a 1×1 pointwise convolution, followed by a shared batch normalization layer. Using a single shared BN rather than per-branch BN reduces buffer memory on MCU platforms.

The two MSME stages progressively halve spatial resolution while expanding channels, going from 32×32×24 to 16×16×32, then to 8×8×48. The resulting output ($F_{m}\in R^{C_{m}\times 8\times 8}$) has $C_{m}=48$, and the full MSME costs approximately 9376 parameters.

### 3.5. Cross-Domain Diagnostic Gate (CDDG)

#### 3.5.1. CDDG Motivation

Stain-colour and morphological features are complementary, yet their relative importance varies from sample to sample. For cells with strong and uniform staining, colour alone is typically sufficient for accurate discrimination. For cells with faint or uneven staining, a common issue in field samples, morphological structure must take over as the primary basis for judgment. An adaptive gating mechanism that learns to modulate each stream’s contribution effectively mimics the diagnostic reasoning that expert microscopists apply during examination.

#### 3.5.2. CDDG Design

Given stain features ($F_{s}$) and morphology features ($F_{m}$) (spatially aligned to 8×8), CDDG computes a channel-wise gating vector through global average pooling and a lightweight, fully connected bottleneck:

(2) $$g=\sigma \left(W_{2}\cdot \mathrm{ReLU}\left(W_{1}\cdot \left[\mathrm{GAP}\left(F_{s}\right);\mathrm{GAP}\left(F_{m}\right)\right]\right)\right)$$

where $W_{1}\in R^{d\times (C_{s}+C_{m})}$ and $W_{2}\in R^{(C_{s}+C_{m})\times d}$ are FC weights with bottleneck dimension of $d=(C_{s}+C_{m})/4=18$ and σ is the sigmoid function.

The gate vector (g) is split into $g_{s}$ and $g_{m}$ and applied element-wise to each stream:

(3) $$F_{\mathrm{fused}}=\left[g_{s}⊙F_{s}\;;\;g_{m}⊙F_{m}\right]$$

Unlike SE-Net [19], which applies channel attention within a single feature map, CDDG performs cross-domain gating: it uses information from both streams to modulate each stream’s contribution. This has a clear diagnostic interpretation: the gate learns which signal domain is more reliable for each specific input.

### 3.6. Prototype Cosine Head (Variant Used as a Leakage Case Study)

We probe whether slide leakage can produce an apparent architectural improvement, not just an inflated headline. To this end, we define a variant, MalariaNet-P, in which only the final classifier is replaced. The stem, SCFE, MSME, CDDG and shared head are inherited unchanged, so the inference graph and MCU cost are essentially identical: 21,083 vs. 21,085 parameters. The motivation is the standard one for metric classifiers. An unconstrained affine map can transfer poorly under domain shift because feature scale and offset drift, while the direction stays discriminative. The head maintains two learnable class prototypes ($p_{0},p_{1}\in R^{64}$); the logit for class *c* is

(4) $$z_{c}=s\cdot \frac{f^{⊤}p_{c}}{{∥f∥}_{2}\;{∥p_{c}∥}_{2}},$$

a scaled cosine similarity. This is the nearest-class-mean or prototypical classifier [20], with no angular margin and *s* representing a single learnable scalar. Under per-cell training, this variant appears to provide a large, seed-stable cross-site robustness gain, a textbook “contribution”. We deliberately retain it not as a proposed method but as the clearest case study, showing that this apparent gain is a training-time leakage artefact that vanishes under leakage-free evaluation (Section 4.8).

### 3.7. Multi-Task Head

The fused features ($C_{s}+C_{m}=72$ channels at 8×8) pass through a shared compact head—1×1 pointwise convolution (72 to 48), depthwise 3×3 convolution with a stride of 2 (8×8 to 4×4), 1×1 pointwise convolution (48 to 64), and global average pooling—producing a 64-dimensional feature vector. All convolutions use BN and ReLU6. This shared feature vector feeds two lightweight task heads:

- Classification head: A single FC layer (64 to 2) for infection detection (parasitized vs. uninfected);
- Burden estimation head: A single FC layer (64 to 1) with sigmoid activation, predicting a continuous parasite burden score in [0,1]. The burden score is auto-generated from image analysis as the normalized dark-stain pixel ratio within each cell, serving as a proxy for parasite load.

The multi-task loss combines both objectives:

(5) $$L=\alpha \cdot L_{\mathrm{cls}}+\left(1-\alpha \right)\cdot L_{\mathrm{reg}}$$

where $L_{\mathrm{cls}}$ is cross-entropy with label smoothing of 0.1, $L_{\mathrm{reg}}$ is MSE loss for burden estimation, and α=0.7. The burden estimation head adds only 65 parameters (<0.3% overhead), enabling multi-task capability at negligible cost.

### 3.8. Complexity Analysis

Table 1 summarizes the parameter distribution across MalariaNet’s components.

The total model size is 85.3 KB in FP32 format and 23.5 KB after INT8 quantization of the weights, as confirmed by on-device measurement. Both fit well within the STM32H7’s 2 MB Flash constraint. The numerically faithful forward keeps fp32 activations to match the quantization scheme of the reported accuracy. It uses a measured 489 KB of activation SRAM at the 128×128 evaluation resolution (Section 4), which is comfortably within the available 1 MB.

With the established architecture and parameter budget, the next section uses this design as a controlled testbed. It defines the per-cell versus slide-disjoint protocols and quantifies how slide leakage inflates accuracy and distorts per-module and variant-level conclusions, then characterises what the design delivers: a leakage-free, on-device-deployable compact baseline.

## 4. Experiments

This section evaluates MalariaNet along four axes, all under the leakage-free, slide-disjoint protocol unless explicitly labelled per-cell.

1. How much the per-cell protocol inflates the headline accuracy relative to slide-disjoint evaluation across all models.
2. Whether the per-module ablation gains reported under per-cell splitting survive leakage-free evaluation; they do not.
3. On-device deployment measurements on STM32H743 hardware, which are split-independent.
4. Characterising, not claiming, the cross-dataset failure mode and the per-cell-only robustness illustration.

### 4.1. Experimental Setup

#### 4.1.1. Dataset

**Figure 3:**
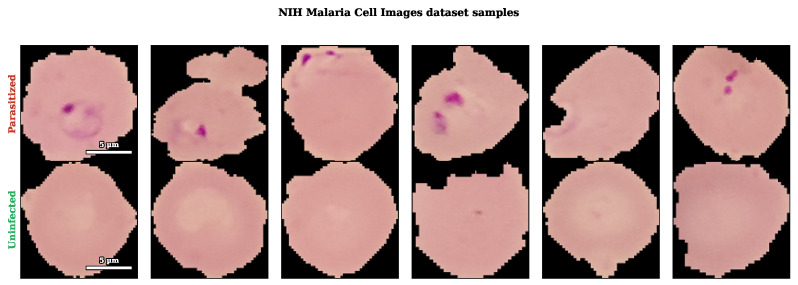
NIH Malaria dataset samples (resized to 128×128). Top: parasitized. Bottom: uninfected. The white bar is an approximate ∼5 μm scale reference; because cells are individually segmented and resized, the absolute scale varies between panels. Colours are the native Giemsa-stain appearance, not encoded values.

We evaluate MalariaNet on the NIH Malaria Cell Images Dataset, a publicly available benchmark containing 27,558 cell images equally divided between parasitized (13,779) and uninfected (13,779) classes. All images are resized to 128×128 pixels. Figure 3 shows representative examples.

#### 4.1.2. Evaluation Protocol: Per-Cell vs. Slide-Disjoint

The 27,558 NIH cell images are not independent. They were segmented from the thin smears of roughly 200 patients/slides, and the originating slide is recoverable from the filename (a leading C〈digits〉 clinic/case identifier, parsable for 100% of the images in both classes). Grouping by this C number yields 200 unique groups covering all 27,558 cells, with the largest holding 2.5% of the cells. The finer C〈digits〉P〈digits〉 slide token parses only 86.5% of file names, so we group at the C-number case level. This level is at or above slide granularity and therefore never splits one case across the training and test sets. The protocol used almost universally in the NIH malaria CNN literature and in the first version of this work is a per-cell randomly stratified split at 70/15/15. It places cells from the same slide into training, validation and test sets. Because intra-slide cells share a staining batch, illumination, focus and background, slide identity leaks across the split and optimistically biases every reported metric.

We therefore evaluate under two protocols and report both. The first is per-cell, the literature-standard, optimistic protocol, retained only for comparability with prior work. The second is slide-disjoint, a leakage-free GroupShuffleSplit on the slide identifier. It splits 70/15/15 by slide group, ≈140/30/30 of the ≈200 slides, with no slide appearing in more than one split and disjointness asserted in code. Unless explicitly labelled per-cell, all headline, ablation and variant results in this paper are reported under the rigorous slide-disjoint protocol with three seeds: 42, 123 and 2024. Quantifying the gap between the two protocols on this benchmark is one of the contributions of this paper, as discussed in Section 5.

#### 4.1.3. Compared Methods

We compare MalariaNet against two categories of models. The first category is large baseline models, representing standard deep learning approaches:

- ResNet-18 [21]: A widely used standard CNN with 11.18 M parameters.
- MobileNetV2 [9]: 2.23 M parameters, designed for mobile deployment.
- EfficientNet-B0 [11]: 4.01 M parameters, NAS-optimized for efficiency.

The second category is MCU-comparable lightweight models for fair comparison at a similar parameter scale:

- Tiny-MobileNetV2 (width = 0.1): 89 K parameters, MobileNetV2 scaled-down with width a multiplier of 0.1.
- SingleStream: 8 K parameters, a single-stream depthwise-separable CNN with the same computational budget as MalariaNet but without domain-specific design.

#### 4.1.4. Training Details

All models are trained with the Adam optimizer (lr = 0.001; weight decay = $1\times 10^{-4}$), a cosine annealing learning-rate scheduler with 5-epoch warm-up, a batch size of 64, and early stopping (patience = 15). Large baseline models use ImageNet-pretrained weights, which gives them a significant advantage over transfer learning. MalariaNet and its variants are trained from scratch without pretraining, so they start at a disadvantage relative to pretrained baselines. Data augmentation includes random horizontal/vertical flipping, rotation (±15), colour jitter, and random affine transformations. To ensure statistical reliability, MalariaNet is trained with three random seeds (42, 123, and 2024), and results are reported as mean ± standard deviation. All experiments are conducted on a single NVIDIA RTX A6000 GPU.

#### 4.1.5. Evaluation Metrics

We report accuracy, sensitivity (recall for the parasitized class), specificity (recall for the uninfected class), F1 score, and AUC-ROC. For deployment analysis, we report model size in KB, parameter count, and FLOPs. Due to the balanced 50/50 class split, accuracy and F1 score are numerically similar; we primarily report accuracy for conciseness.

### 4.2. Main Results Under the Literature-Standard Per-Cell Protocol

We first report results under the per-cell protocol that prior work on this benchmark has used, so our numbers are directly comparable to the literature. Section 4.4 shows what happens to these same numbers under leakage-free, slide-disjoint evaluation. The per-cell figures in Table 2 should be read as optimistic upper bounds, not as the operating accuracy of a deployed device.

Two features of Table 2 require careful reading. First, the primary observation is the unanimous direction: every one of the eight models has Δ<0, so the inflation is a property of the per-cell protocol, not of one architecture. The individual per-model magnitudes are mostly within the slide-disjoint seed standard deviation—for example, MalariaNet’s −1.45 against its own ±1.02—so we do not rest the claim on any single Δ. The eight models also share the same dataset and leaked slides, so their Δ values are not statistically independent, and we report no sign-test *p*-value. We quantify the test-set uncertainty at the headline operating point in three ways. A per-cell Wilson 95% interval on the per-seed slide-disjoint test splits, which hold 3320 to 4719 cells across 30 slide groups, is only ±0.6 pp. This interval assumes cell independence and is anti-conservative because cells cluster within slides. A slide-level cluster (block) bootstrap that, instead, resamples all test slides (20,000 resamples) widens the 95% interval to ±1.2 pp at seeds 42 and 123 and to ±3.3 pp at seed 2024, a design effect of 4 to 27 over the naive binomial variance. For the binding uncertainty we report, the cross-seed standard deviation of ±1.02 pp is consistent with these clustered intervals and exceeds the naive one, and the −1.45 pp gap falls within it. The quantitative strength is the unanimous sign, together with the collapse of every design conclusion in Section 4.4 and Section 4.8, not the headline magnitude. Second, there is an apparent differential: the large ImageNet-pretrained backbones move less (−0.25 to −0.47 pp) than the compact from-scratch models (−0.86 to −1.45 pp). This would suggest per-cell evaluation specifically flatters the compact-model regime this literature targets. This pattern is confounded with the from-scratch vs. ImageNet-pretrained distinction, so we ran a controlled experiment: the three large baselines retrained from scratch under both protocols with three seeds and an identical recipe. The result is mixed, and we report it without selection. ResNet-18 and MobileNetV2 move much more once their ImageNet initialisation is removed. Their leakage Δ grows from −0.25 to −0.75 and from −0.39 to −0.97 pp, into the compact-model range. EfficientNet-B0, however, is essentially unchanged from −0.47 to −0.56 pp and within its ±0.79 seed deviation. The protective effect of ImageNet pretraining against slide leakage is therefore real but architecture-dependent, not a universal law. It accounts for two of three large backbones and not the third. Consequently, we do not treat the compact-versus-large differential as a primary result. The principal claim remains the unanimous inflation and the collapse of every design conclusion. Full from-scratch numbers are summarised in Section 5. Third, the consequence for the original claim is robust, regardless of the above. Under per-cell splitting, MalariaNet at 97.06% appears to match MobileNetV2 at 96.90% with 100× fewer parameters. Under leakage-free evaluation, MalariaNet at 95.61% sits ≈1 pp below every larger baseline—namely, EfficientNet at 96.75, ShuffleNet at 96.56 and MobileNetV2 at 96.51. The “compact model matches large models” narrative was, itself, a leakage artefact. What remains true and protocol-independent is the deployment envelope: MalariaNet is the only entry simultaneously satisfying <200 KB and <10 M FLOPs (Section 4.5). The positioning is therefore that of a radically compact, MCU-deployable detector that trades ≈1 pp accuracy against the best larger baselines under rigorous evaluation, not a model that matches them.

**Figure 4:**
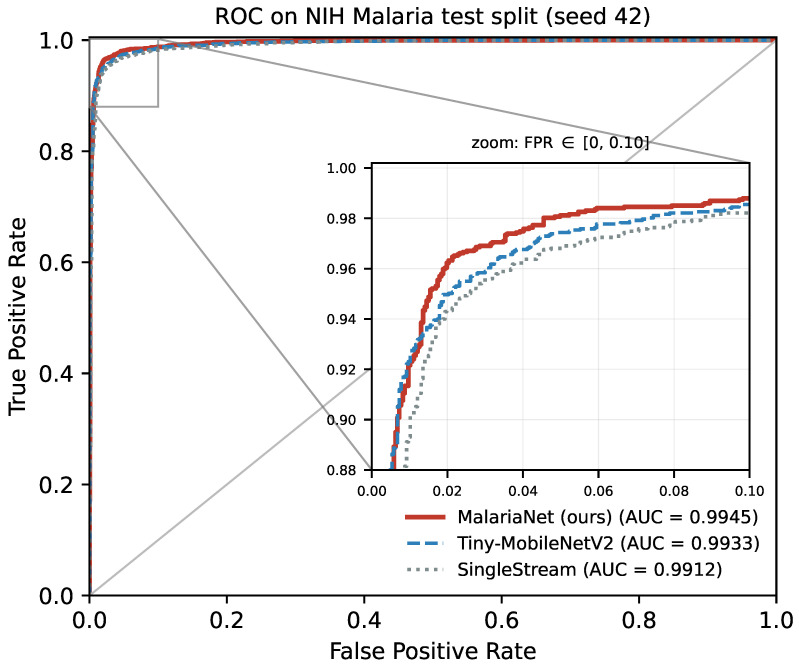
ROC on the NIH test split with the per-cell protocol (optimistic, seed 42), retained for comparability with prior work. Per-cell AUCs are inflated relative to the slide-disjoint values (MalariaNet, 0.988±0.003; Table 3); the small inter-model gaps shown here do not survive leakage-free evaluation and are not used to claim superiority. The grey diagonal marks chance (AUC 0.5); the inset zooms into the low-false-positive region (FPR ≤0.10), where the otherwise-overlapping curves separate.

Figure 4 shows per-cell optimistic ROC curves on the seed-42 NIH test split for MalariaNet and the two MCU-deployable baselines, provided for comparability with prior work that reports per-cell ROC. Consistent with the rest of this section, the per-cell separation is small and should not be over-interpreted. Under slide-disjoint evaluation, MalariaNet’s AUC is 0.988±0.003, as reported in Table 3, which is below the ≈0.994 the per-cell protocol suggests, and the variant-to-variant ordering is not stable across protocols. We include the curve only to characterise the operating region of a high true-positive rate at a low false-positive rate relevant to a screening device, not as evidence of architectural superiority.

### 4.3. Clinical Decision Metrics Under Endemic Prevalence

Accuracy at the balanced 50/50 NIH test split overstates real-world diagnostic value. Malaria prevalence in field settings ranges from <1% in low-transmission regions to 10–20% during peak season in sub-Saharan Africa [1]. We therefore re-express the slide-disjoint 3-seed operating point (sensitivity = 93.94%; specificity = 97.41%; Table 3) as the positive predictive value (PPV) and negative predictive value (NPV) at clinically realistic prevalence rates. We use the leakage-free operating point deliberately: PPV/NPV at the inflated per-cell sensitivity would, itself, be optimistic.

Three operational implications follow from Table 4. First, in low-prevalence elimination settings below 1%, PPV is only 15–27%, so most “positive” device outputs require confirmatory microscopy or RDT before treatment. This is consistent with WHO screening-test guidance and is not unique to lightweight models. According the Bayes theorem, the same effect arises for any ∼94%/97% sensitivity and specificity test under low prevalence. Second, in mesoendemic to hyperendemic settings with 5–20% prevalence, the realistic deployment target for community health workers in malaria-endemic Africa and South Asia, PPV reaches 66–90%. This makes the device clinically actionable for triage and case prioritisation. Third, NPV stays ≥99% across the 0.5–10% prevalence band and 98.5% at 20%, so a negative result reliably rules out malaria, the operating mode in which a screening tool is most valuable. We position MalariaNet as a triage or mass-screening aid rather than a stand-alone diagnostic, with positive results referred to confirmatory testing as recommended for any periphery-level diagnostic tool.

These predictive values are computed at the in-domain (same-source, leakage-free), slide-disjoint operating point and are therefore an upper bound. Predictive value depends only on sensitivity, specificity and prevalence, not on accuracy; under a cross-laboratory shift, where both sensitivity and specificity fall (Section 4.7), they degrade accordingly. At the MP-IDB cross-laboratory operating point (sensitivity, 74.0%; specificity, 82.7%) the same Bayesian calculation give PPVs of about 18%, 32% and 52% at 5%, 10% and 20% prevalence, with NPVs of about 98.4%, 96.6% and 93%. Therefore, the 66–90% PPV and ≥99% NPV reported above characterise in-domain operation only and should not be read as cross-site performance.

### 4.4. Ablation Study Under Slide-Disjoint Evaluation

We ablate each module under the rigorous slide-disjoint protocol of Section 4.1.2: the NIH cells are split by patient or slide group so that no slide contributes images to more than one among the training, validation and tests. Every configuration is retrained from scratch over three seeds (42, 123, and 2024), with all other hyperparameters fixed. Results are presented in Table 3. ΔAcc is the full-model accuracy minus the variant.

Under leakage-free evaluation and reported without selection:

1. Per-module gains do not survive rigorous evaluation. The full model (95.61 ± 1.02%) differs from SingleStream by only +0.24 pp and from Uniform-scale MSME by +0.07 pp and is, in fact, marginally lower than w/o SCFE (−0.03 pp) and w/o CDDG (−0.08 pp). Every difference is far smaller than the ≈1 pp seed-to-seed standard deviation, and none is statistically distinguishable. No contrast attains significance, even uncorrected, so multiple-comparison control, such as via Bonferroni or Holm correction, would only widen these intervals and reinforce the null hypothesis. Such correction is therefore not the binding consideration here. We consequently do not claim individually validated per-module contributions.
2. The per-cell protocol inflated both the headline and every ablation delta. Under the optimistic per-cell split, the same architecture reported 97.06% with module gains of +0.78/+0.38/+0.56/+0.37 pp. Under slide-disjoint evaluation, the headline falls by 1.45 pp, and the module gains collapse to within noise and partly invert. This quantifies how strongly slide leakage flatters NIH malaria results. To the best of our knowledge, this methodological caution has not been quantified for this benchmark, and we regard it as a contribution in its own right, as discussed in Section 5.
3. What the architecture does provide is a compact 21 K-parameter detector that remains at ≈95.6% under rigorous evaluation while fitting the STM32H7 envelope, as quantified in Section 4.5. Consider prototype-cosine variant MalariaNet-P. Its apparent +7 pp cross-site gain under per-cell training is shown Section 4.8 to be a per-cell-training leakage artefact, collapsing to +1.8 pp within ±5 pp seed noise under slide-disjoint training. We therefore do not claim it as a robustness contribution. We retain it only as a case study of leakage producing an apparent innovation. We position the stain–morphology decomposition as an interpretable, parameter-frugal design, not as a set of separately significant ablation gains.

### 4.5. Deployment Analysis

Table 5 summarizes the deployment feasibility on the STM32H7 series (Cortex-M7 up to 480 MHz, 2 MB Flash, 1 MB RAM; our test board runs at 400 MHz).

MalariaNet at 85.3 KB in FP32 or 23.5 KB in INT8 weights comfortably fits within the STM32H7’s Flash memory, leaving ample space for firmware, runtime, and application code. The numerically faithful forward stores the convolutional and linear weights as int8 and keeps batch-norm and activations in fp32, so it reproduces exactly the quantization scheme under which the 95.61% accuracy is measured. This forward uses 489 KB of activation SRAM at the 128×128 evaluation resolution, within the 1 MB on-chip budget.

The 200 KB model-size threshold is derived from a practical STM32H7 Flash budget: 2 MB total minus ∼500 KB for firmware and HAL drivers, ∼300 KB for runtime, and ∼1 MB reserved for application code and image buffers, leaving approximately 200 KB for the neural network model.

For on-device validation, we flashed a numerically correct INT8-weight forward (convolutional and linear weights stored as symmetric per-tensor int8 and dequantized at use, batch-norm and activations in fp32) onto an STM32H743IIT6 development board (Cortex-M7 @ 400 MHz, I-Cache and D-Cache enabled) and timed it with the DWT cycle counter. The on-device output matches the off-device reference to a maximum logit deviation of $1.8\times 10^{-4}$. This confirms that the deployed kernel computes the same classification as the evaluated model rather than a workload proxy. Table 6 reports the measured cost.

At the 128×128 evaluation resolution, the faithful forward runs in 816 ms (1.23 FPS, mean over 10 iterations, range 816.0–816.3 ms, deterministic to <0.3 ms). This is a triage rate rather than real-time video: it classifies roughly 74 cells per minute, which is still far faster than manual expert microscopy at 20–30 min per slide. This is the cost of the reference kernel. The implementation is an unoptimised, hand-written forward with no CMSIS-DSP or CMSIS-NN acceleration, and it keeps fp32 activations to match the quantization scheme of the reported accuracy. A vendor-optimised kernel or a full int8-activation pipeline would reduce both the latency and the 489 KB activation footprint. That gain would come at the cost of adopting a quantization scheme whose accuracy would require separate validation, which we therefore do not claim here. The model weights occupy only 23.5 KB of the 2 MB Flash (1.1%).

Quantization is near-lossless. We applied the deployed INT8 scheme, symmetric per-tensor 8-bit quantization of every convolutional and linear weight in the CMSIS-NN or TensorFlow Lite Micro int8 weight format and re-evaluated on the NIH test split across three trained checkpoints. The change in test accuracy from FP32 to INT8 is +0.01±0.05 pp, i.e., within seed-to-seed variance and statistically indistinguishable from zero. The 23.5 KB INT8 model therefore preserves the full-precision diagnostic accuracy. Thus, all accuracy results reported in this paper hold for the actually deployed integer model, not only for its floating-point counterpart.

### 4.6. Comparison with Published Methods

Table 7 compares MalariaNet with recently published methods on the NIH Malaria dataset.

This comparison must be read with care, and it motivates our central point. All listed prior methods report per-cell accuracy on NIH, and none, to the best of our knowledge, evaluates a slide-disjoint configuration. At the per-cell operating point, MalariaNet at 97.06% is competitive with these methods, reporting 95.0 to 99.2%, corresponding to 6× to over 4000× fewer parameters. That per-cell figure is optimistic for every row, not only ours. Under the leakage-free, slide-disjoint protocol, MalariaNet operates at 95.61±1.02%. The corresponding slide-disjoint numbers for the other entries are not available because those works did not run the protocol. We therefore do not claim to beat prior methods on accuracy. We claim that the field accuracies, including earlier versions of our own, are reported under a leaky protocol, and we provide, to the best of our knowledge, the first slide-disjoint, on-device reference. Among all entries, MalariaNet is the only one explicitly designed and validated for MCU constraints: 23.5 KB INT8 weights in Flash and a 489 KB activation footprint that fits the STM32H743’s 1 MB SRAM, a property independent of the evaluation split.

### 4.7. External-Dataset Validation: Zero-Shot Transfer to BBBC041

**Figure 5:**
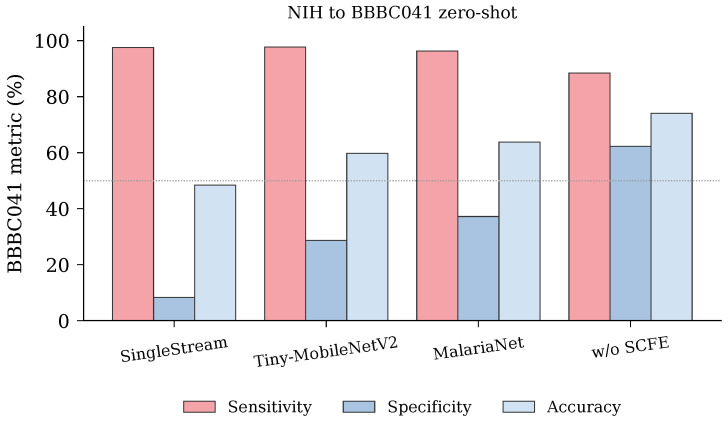
Zero-shot NIH to BBBC041 transfer (per-cell-trained models, no fine-tuning). All models keep high sensitivity but collapse in specificity off-domain. The apparent left-to-right ordering is a per-cell training pattern that does not survive slide-disjoint training (Section 4.8); we draw no architectural conclusion from it. The robust message is that no compact model transfers acceptably to the unseen species without target-site adaptation. The dashed horizontal line marks the 50% chance level.

To probe generalisation beyond the single NIH source, we validate on an independent dataset acquired at a different laboratory. We test NIH-trained zero-shot models on BBBC041 [23], a Broad Institute *P. vivax* dataset with different staining and scanner setups. It comprises 5452 extracted cells (2452 parasitized and 3000 uninfected) with no fine-tuning. This is a deliberately demanding external test: it combines a species shift, i.e., *P. falciparum* to *P. vivax*, with a staining and scanner shift. We complement it below with a same-species cross-laboratory test on MP-IDB [24] that isolates the laboratory shift from the species shift; a broader multi-centre, multi-patient validation remains a subject for future work (Section 5). Table 8 and Figure 5 report the per-cell-trained models for comparability with the original analysis. Caveat: The prototype-cosine case study of Section 4.8 showed that cross-site rankings obtained from per-cell-trained models do not survive slide-disjoint training. The same caveat applies here, so the ordering below should be read as indicative of the difficulty of the *P. falciparum* to *P. vivax* shift, not as evidence of architectural superiority.

All models degrade sharply off-domain, as expected, given the species, staining and scanner shifts. According to the apparent ordering, MalariaNet is above the task-agnostic compact baselines and w/o-SCFE is the highest, mirroring exactly the pattern that, for the prototype-cosine variant, we showed to be a per-cell training artefact (Section 4.8). We therefore draw no architectural conclusion from this table. Its only robust message is that none of these compact models transfers acceptably to an unseen species without target-site adaptation, consistent with the limitations discussed in Section 5. On the fixed 5452-cell BBBC041 set, these off-domain rates carry tight binomial intervals. For example, MalariaNet specificity is 37.2% (Wilson 95% [35.5,38.9], n=3000), and the w/o-SCFE variant has a sensitivity of 88.4% ([87.1,89.6], n=2452) and a specificity of 62.3% ([60.5,64.0], with n=3000). Thus, the specificity collapse is well outside of sampling noise and is a genuine domain-shift effect, not a small-sample artefact.

An unexpected outcome is that the w/o SCFE variant achieves the highest cross-dataset accuracy of 74.03%. The fixed Giemsa projection vectors, while effective for the NIH dataset’s specific staining conditions, may over-specialize to a particular colour distribution. This points to data-driven calibration of the stain projection—rather than fixed vectors—as a route to improving cross-domain robustness. We discuss this important direction further in Section 5.

#### Same-Species Cross-Laboratory Validation (MP-IDB)

The BBBC041 test discussed above couples a laboratory shift with a species shift. To isolate the laboratory effect, we add a same-species external test on MP-IDB [24], a *P. falciparum* thin-smear dataset acquired at a different laboratory, with a different microscope and staining procedure, from 104 expert-annotated fields. Using the dataset’s expert parasite ground truth for infected cells and automated red-blood-cell segmentation for uninfected cells, with cells overlapping a parasite or a leukocyte excluded, we extracted 1185 parasitized and 2000 uninfected single-cell crops, which were constructed exactly as for the NIH and BBBC041 cell sets. We evaluate the deployed slide-disjoint MalariaNet zero-shot configuration with no fine-tuning over the same three seeds (Table 9).

The contrast is informative. Under the pure *P. falciparum* laboratory shift, MalariaNet keeps a usable operating point: accuracy, 79.5±2.9%; sensitivity, 74.0±8.3%; specificity 82.7±5.8%; and AUC, 0.865±0.029. This is a clear drop from the in-domain, slide-disjoint accuracy of ≈95.6% but far from collapse, and the decision threshold stays calibrated. The ≈16 pp gap reflects concrete differences between the two sources rather than a failure of the detector. The NIH cells are provider-segmented crops from Chittagong Medical College Hospital (Bangladesh), whereas the MP-IDB cells are segmented by us from a European laboratory’s 100× full-field images captured with a different camera and Giemsa realisation, so stain colour, optics, scale and cell framing all shift between the training and test sets. Because the morphology of the *P. falciparum* parasite is nonetheless shared, ranking is largely preserved (AUC of 0.865), and the operating point stays usable, which is exactly what separates this same-species shift from the cross-species case below. Adding the species shift to *P. vivax* (BBBC041, same slide-disjoint model and seeds) instead collapses specificity to 18.0±12.8% at an accuracy of 53.2±5.2%. A same-species change of laboratory therefore degrades the detector gracefully, whereas a change of species breaks its operating point. We read MP-IDB as encouraging but partial evidence: it is a single additional laboratory, and the inputs are well-formed cell crops rather than end-to-end field captures. A further caveat concerns the magnitude of the drop. The NIH cells we train on are segmented by the data providers, whereas for MP-IDB, we use the dataset’s expert parasite ground truth for the infected cells but segment the uninfected cells ourselves, so the result conflates a genuine laboratory shift with a difference in cell isolation that cannot be fully separated with the available data, since any external *P. falciparum* set requires our own segmentation. One internal check using only the present numbers bears on this: if our segmentation of the uninfected cells had introduced poor crops, specificity should have suffered most, yet specificity (82.7%) exceeds the sensitivity on the provider-annotated infected cells (74.0%). This does not support segmentation quality as the primary driver of the drop, though it cannot exclude a contribution. We therefore treat MP-IDB as directional evidence of graceful same-species degradation, not as a precise measure of the domain-shift magnitude, and a broader same-species validation across more laboratories, scanners and patient populations, ideally with a segmentation-effect control, remains necessary (Section 5).

### 4.8. Case Study: Leakage Fabricates an Apparent “Robustness Variant”

A central illustration of how slide leakage produces not just numbers but apparent innovations is the MalariaNet-P prototype-cosine variant (Section 3.6), which changes only the final classifier (21,083 vs. 21,085 parameters; identical MCU inference cost). It was originally adopted because, under per-cell training, it appeared to deliver a large, consistent cross-site robustness gain. Table 10 shows what happens when the same variant and baseline are retrained under the leakage-free, slide-disjoint protocol and evaluated identically. BBBC041 is a separate *P. vivax* dataset, so cross-site evaluation is intrinsically leak-free; the variable here is the training protocol.

The contrast is the point. Under per-cell training, the variant shows +7.0 pp BBBC041 accuracy from 63.79 to 70.82 with tight variance. This is exactly the profile of a publishable “robustness contribution”, and, indeed, an earlier version of this work presented it as one. Under leakage-free, slide-disjoint training, the same comparison yields 53.23±5.23 vs. 55.04±5.69%, a +1.8 pp difference that is smaller than its own ±5.5 pp seed noise and changes sign across seeds, with the variant being worse at seed 42. The in-domain difference is likewise null under rigorous evaluation (95.61±1.02 vs. 95.69±0.82%). We therefore do not claim MalariaNet-P as a robustness contribution. We report it as the clearest case study of this paper’s primary finding. Slide leakage in training does not merely inflate a headline by a point or two. It can manufacture a plausible, internally consistent, seed-stable “architectural innovation” that evaporates under leakage-free evaluation. This is precisely why slide-disjoint training and testing must be standard for this benchmark.

### 4.9. A Parasite-Burden Head at Negligible Deployment Cost

A parasite-burden regression head can be attached to MalariaNet at negligible cost: it adds 65 parameters, which is below 0.3% of the model, so the multi-task option leaves the microcontroller memory and latency budget unchanged. We do not report its accuracy or burden correlation here because the head was trained and evaluated only under the per-cell protocol that this paper argues is unreliable; a leakage-free multi-task re-evaluation is left to future work. Therefore, the only protocol-independent statement is this structural one.

### 4.10. Robustness to Imaging Interference Across Compared Models

**Figure 6:**
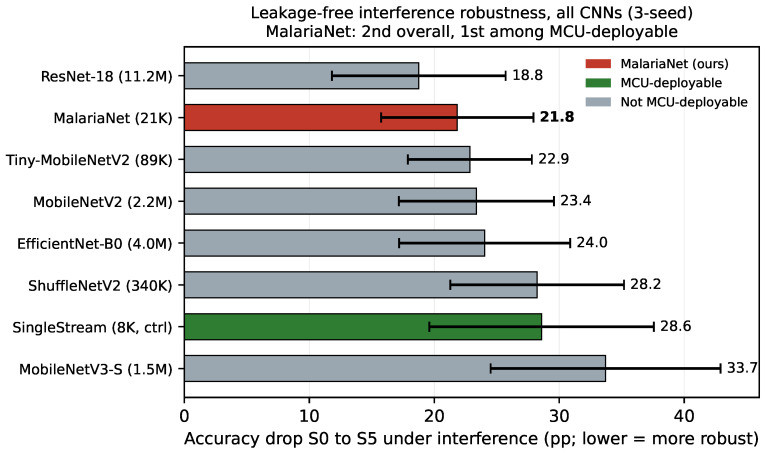
Leakage-free interference robustness across all eight CNNs (slide-disjoint, 3-seed; error bars = seed std). MalariaNet (red) is the second most robust overall and the most robust MCU-deployable model; SingleStream (the deployable control) is among the least robust.

The portable-microscope deployment scenario is defined by degraded imaging artefacts such as colour cast, noise, blur and low resolution, so resilience to interference matters more than clean accuracy. We therefore evaluate it rigorously. Every model is the slide-disjoint-trained checkpoint with three seeds, evaluated on the slide-disjoint test split under a six-level interference sweep (S0 = clean to S5 = heavy), with severity representing the probability each PIDM-style degradation factor is applied. Unlike the per-cell, single-seed stain analysis in the first version of this work, this is a leakage-free, multi-seed, all-CNN head-to-head analysis. Table 11 ranks all eight models by accuracy drop from S0 to S5, and Figure 6 visualises the results.

This is, in contrast to the collapsed per-module ablations, a genuine finding that survives leakage-free evaluation. Three readings follow. First, MalariaNet, with a 21.84±6.10 pp drop, is more interference-robust than EfficientNet-B0, MobileNetV2, MobileNetV3, ShuffleNetV2 and the SingleStream control. It is beaten only by ResNet-18 at 18.76 pp, which is 530× larger and cannot be deployed. Among the two MCU-deployable models, MalariaNet is far more robust than SingleStream (21.84 vs. 28.59 pp) and negative-signed in all three seeds, with the gap widening monotonically from +0.2 pp at S0 to +7.0 pp at S5. Second, we state the caveats plainly. The per-seed std is large (±5–9 pp), so adjacent ranks such as MalariaNet vs. Tiny-MV2 are within noise. The robust claim is the cluster position (top two or three and clearly above the ShuffleNet, SingleStream and MNv3 group), together with the seed-consistent MalariaNet > SingleStream margin, not a precise ranking. The degradation is simulated, not physically captured. Finally, the clean S0 accuracy gap relative to larger models (≈1 pp) is unchanged, which is a separate, deployment-critical resilience property, not a clean-accuracy claim. Third, we do not attribute it to any single module, since the leakage-free ablation shows none is individually significant. The statement we make is that the compact architecture, as a whole, is markedly more interference-robust than the naive single-stream control and than most larger CNNs. This is exactly the property the portable-microscope scenario requires.

### 4.11. Knowledge Distillation: A Partial Mitigation of the Pretraining Gap

The ≈1 pp clean-accuracy gap relative to the larger baselines is largely the from-scratch-vs-ImageNet-pretrained distinction, as discussed in Section 5. The compact models that train from scratch—namely, MalariaNet, SingleStream and Tiny-MV2—cluster ≈1 pp below the pretrained backbones. Because a 21 K custom architecture cannot be meaningfully ImageNet-pretrained, we test whether knowledge distillation can transfer that advantage indirectly: the leakage-free EfficientNet-B0 teacher, the slide-disjoint protocol at 96.75%, distilled into the fixed 21 K student with a loss of $\left(1-\alpha \right)\;\mathrm{CE}+\alpha T^{2}\;\mathrm{KL}$ and T=4. This is training time only. The deployed inference graph is byte-identical to MalariaNet, so the MCU envelope is unchanged.

Under three-seed, slide-disjoint distillation with α=0.7, the student reaches 95.96±0.68%, versus MalariaNet’s 95.61±1.02%, corresponding to a +0.35 pp mean gain with reduced variance. Because the gain is small, we report it per seed rather than as the mean alone (Table 12). The seed-paired change is −0.09 pp at seed 42, +0.35 pp at seed 123 and +0.78 pp at seed 2024, so it is non-positive at seed 42 and positive at the other two seeds. The mean change is +0.35 pp with a paired-*t* 95% confidence interval of [−0.73,+1.43] pp (p≈0.30) and a paired effect size of Cohen’s $d_{z}\approx 0.80$ whose interval also spans zero at n=3. The gain is therefore not statistically established. Because n=3, this effect size is, itself, unstable, and we do not interpret its magnitude; we report it only for completeness alongside the per-seed values and the confidence interval. With α=0.5/0.7/0.9, the seed-42 student accuracy is 96.60/96.57/96.30%, so the per-seed picture is not an artefact of one α. The reading we adopt is that distillation recovers about one-third of the ≈1.1 pp gap relative to EfficientNet-B0 and reduces seed variance yet leaves the student ≈0.8 pp below the strongest baseline. It does not make the compact model accuracy-competitive with the best larger model. It modestly narrows and stabilises the gap at zero inference cost.

### 4.12. Visualising the Leakage Effect

**Figure 7:**
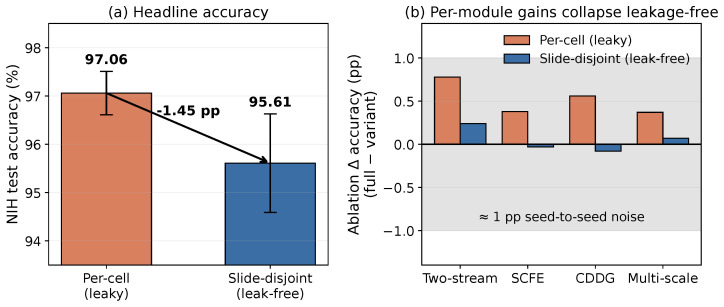
Slide leakage inflates accuracy and distorts ablation conclusions. (**a**) Headline NIH accuracy, per-cell vs. slide-disjoint (3-seed mean ± std). (**b**) Per-module ablation Δ (full − variant): large under per-cell splitting, collapsing into seed noise (grey band) and partly inverting under slide-disjoint evaluation.

The paper’s central result is summarised in Figure 7. Under the leakage-free, slide-disjoint protocol, the headline accuracy falls by 1.45 pp. More importantly, the per-module ablation gains that look substantial under per-cell splitting collapse into the ±0.15 pp noise band, with SCFE and CDDG turning marginally negative. The architectural “story” the per-cell protocol tells is not reproducible under leakage-free evaluation.

**Figure 8:**
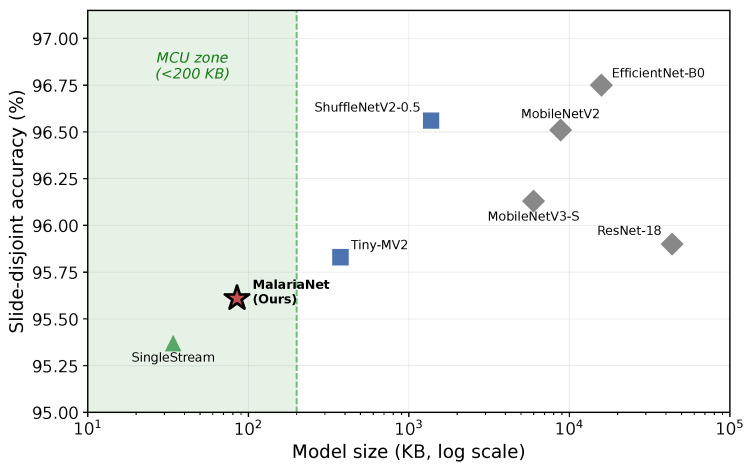
Leakage-free (slide-disjoint) accuracy vs. model size (3-seed mean). Green zone: MCU-deployable (<200 KB). Under rigorous evaluation, MalariaNet is the most accurate model in the deployable zone but sits ≈1 pp below the larger baselines, corresponding to a compactness/accuracy trade-off, not the “matches large models” picture the per-cell protocol produced.

**Figure 9:**
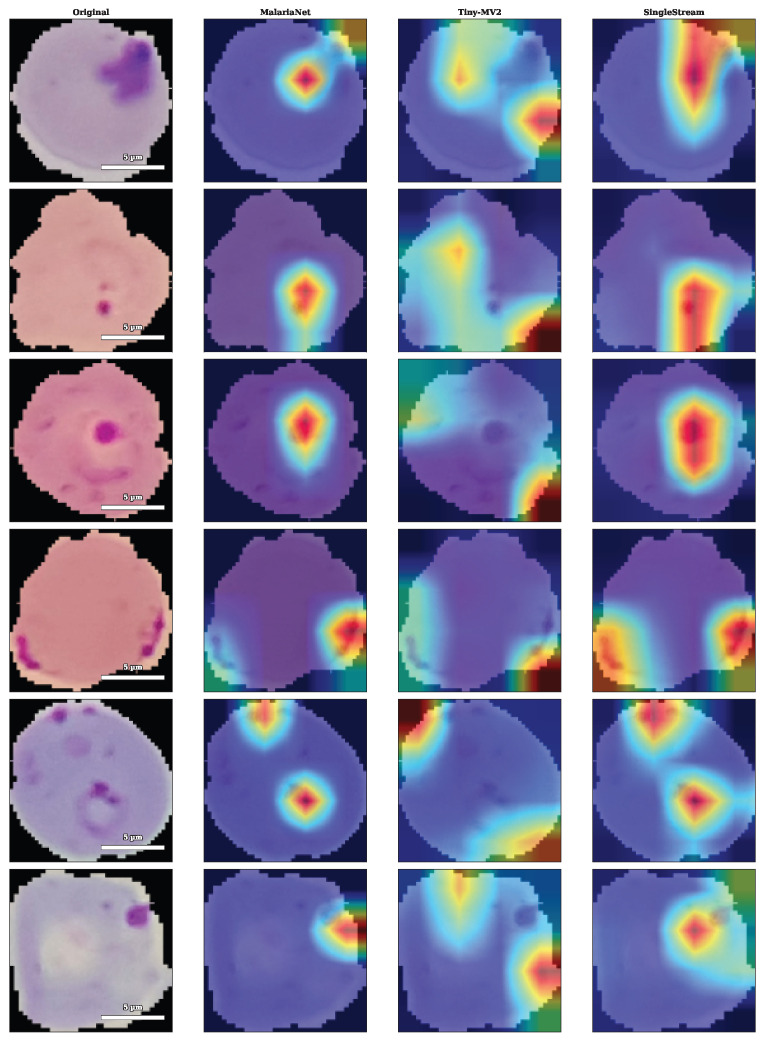
Grad-CAM++ on parasitized cells (qualitative). Columns: original, MalariaNet, Tiny-MV2, and SingleStream; each row is one cell shown across the methods, so a row shares the same scale. All panels are shown at the 128×128 model-input resolution. As in Figure 3, the white bar is an approximate ∼5 μm reference, and the absolute scale varies between rows because cells are individually segmented and resized. Warmer colours indicate higher class-discriminative activation.

**Figure 10:**
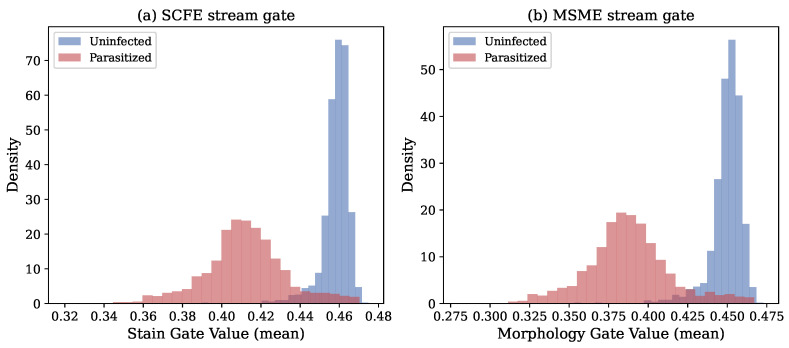
CDDG gate-value distribution (transparency only): the gate takes differentiated values for parasitized vs. uninfected cells, but this does not translate into a measurable accuracy gain under leakage-free evaluation (Section 4.4).

We additionally provide Figure 8, the leakage-free accuracy-size trade-off under slide-disjoint evaluation. It makes the positioning explicit: MalariaNet uniquely fits the MCU zone but at ≈1 pp below the larger baselines. Two qualitative per-cell illustrations are retained for transparency only and support no claim: Figure 9 show Grad-CAM++ [25] attention, and Figure 10 shows the CDDG gate-value distribution. The gate does take differentiated values, yet, per Section 4.4, this confers no measurable accuracy benefit under leakage-free evaluation.

In summary, the experimental evidence supports two claims, not four. First, on the NIH benchmark, the per-cell protocol inflates the headline by 1.45 pp and distorts every per-module ablation conclusion and an apparent cross-site “robustness variant”, as established in Section 4.4 and Section 4.8 and Figure 7. Second, under leakage-free evaluation, a 21 K-parameter detector still reaches ≈95.6% within the STM32H7 envelope, per Table 5 and Table 6, making it a deployable, rigorously benchmarked reference. The next section interprets the leakage finding and its implications for the field.

## 5. Discussion

This section interprets the paper’s primary finding. That finding is that slide leakage inflates accuracy and distorts architectural conclusions on the NIH malaria benchmark. This section then situates the surviving artefact, a deployable compact detector, in the point-of-care context and bounds what remains open.

### 5.1. Slide Leakage and Its Consequences for the MCU Malaria Literature

The NIH Malaria Cell dataset is the de facto benchmark for compact malaria detectors, and per-cell random splitting is its near-universal evaluation protocol. Our results show that this protocol is not merely slightly optimistic: it is actively misleading. Three consequences, all measured here on an identical architecture, are worth separating.

The first is an inflated headline. The same model reports 97.06±0.45% under per-cell splitting and 95.61±1.02% under slide-disjoint evaluation, a 1.45 pp gap. While this gap is of the same order as the ±1.02 slide-disjoint seed standard deviation, its significance does not rest on the headline number alone: the inflation is negative in sign for every one of the eight architectures in Table 2, with per-model Δ ranging from −0.25 to −1.45 pp. We are careful not to over-state this: the eight models share the same dataset and the same leaked slides, so their Δ values are not statistically independent, and a naive $2^{-8}$ sign-test *p*-value would be unjustified. The claim we make is weaker but still strong. Eight independently trained, architecturally diverse models—compact and large, from-scratch and ImageNet-pretrained—all move in the same inflationary direction under per-cell splitting, with no exception. This is difficult to attribute to seed noise, even granting the correlation. Any leaderboard built on per-cell numbers is therefore comparing optimistic upper bounds, not deployable accuracies.

The second consequence is distorted ablation conclusions. Under per-cell splitting, every proposed module showed a clean positive ablation (+0.78/+0.38/+0.56/+0.37 pp). This is the kind of evidence routinely used to justify architectural design. Under slide-disjoint evaluation, these gains collapse into ±1 pp seed noise, and two of them invert, as shown in Table 3. The per-cell protocol did not just inflate a number; it produced a coherent but false story about why the architecture works.

The third consequence is a spurious “robustness variant”.Most notably, the prototype-cosine variant, MalariaNet-P, exhibits a large, seed-stable +7.0 pp cross-site gain under per-cell training, which an earlier version of this work presented as a contribution. Retrained leakage-free, the same comparison gives +1.8 pp within ±5.5 pp seed noise, as reported in Table 10. Leakage can therefore produce an internally consistent, plausible, seed-stable “innovation”. This reflects a protocol flaw rather than a real effect, and we retain the case because it makes the failure mode concrete.

The remedy is cheap: the slide identifier is recoverable from the NIH filenames, so a group-disjoint split costs nothing and removes the leak. We recommend it become the default for this benchmark. The split is fully specified in Section 4.1.2 and reproducible from the public NIH file names alone, with no extra artefact needed. Future compact-malaria work can therefore adopt it directly on a leakage-free footing. The broader caution generalises to any medical-imaging benchmark where many patches share a patient or slide and the standard split is per-patch.

### 5.2. Toward a Point-of-Care Edge Device: Scope and Open System Elements

**Figure 11:**
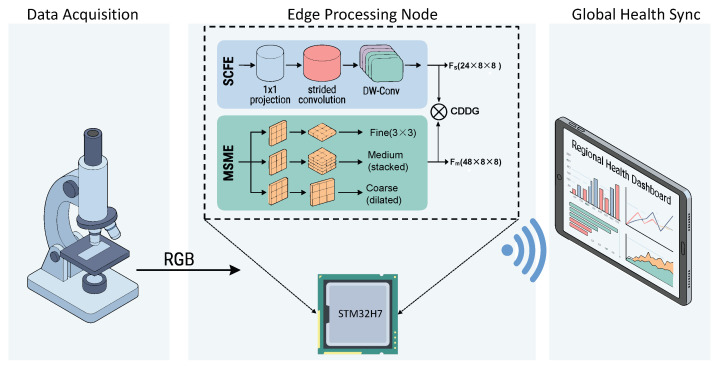
Point-of-care edge deployment concept. Only the on-device single-cell classification stage (STM32H743, measured 816 ms per 128×128 cell) is validated; image acquisition, connectivity and aggregation are shown as concepts. Device power was not measured; the bill of materials is an estimate.

MalariaNet is intended as the inference engine of a point-of-care edge device for malaria cell classification, as illustrated in Figure 11. The surrounding system in Figure 11 is a design concept. We validate only the on-device classification stage for accuracy, latency and memory; the acquisition, connectivity and end-to-end elements described below are context rather than validated capabilities.

In a typical deployment scenario, the system operates as follows. First, a low-cost microscope camera captures blood-smear images. Second, the STM32H7 MCU runs MalariaNet for local inference. It produces infection detection and burden estimation results in a measured 816 ms per 128×128 cell (1.23 FPS). This is the numerically faithful INT8-weight forward on STM32H743 at 400 MHz. Third, results are displayed immediately on a local screen for the healthcare worker. Fourth (and optionally), aggregated diagnostic statistics such as infection rates and temporal trends are transmitted to regional health authorities. Transmission uses low-power wireless protocols such as LoRa or BLE for epidemiological surveillance.

This architecture follows the autonomous edge AI paradigm: the device performs cell-level classification at the point of care without cloud connectivity or centralized infrastructure. Whole-slide diagnosis additionally requires acquisition, focus, detection and aggregation stages that are outside the present scope. We make no claim that any internal module confers a measured accuracy benefit, since the evidence does not support that. The deployable value is a single validated artefact: a 21 K-parameter detector at ≈95.6% leakage-free accuracy within the STM32H7 envelope.

### 5.3. Clinical Significance and Intended Use

The clinically relevant question is not balanced-split accuracy but predictive value at endemic prevalence. From the leakage-free operating point (Section 4.3, Table 4), negative predictive value stays ≥99% across the 0.5–10% prevalence band, so a negative result reliably rules out malaria. Positive predictive value is low below 1% prevalence and rises to 66–90% in the 5–20% range typical of community screening. The intended use is therefore as a triage or mass-screening aid whose positive outputs are referred to confirmatory microscopy or a rapid diagnostic test, consistent with WHO guidance for periphery-level tools, rather than as a stand-alone diagnostic. Because the device classifies pre-segmented single cells, this clinical value is realised only as one stage within a complete workflow that still requires image acquisition, parasite detection and slide-level aggregation. These predictive values use the in-domain operating point and are an upper bound; at the cross-laboratory operating point of Section 4.7, they fall substantially (Section 4.3).

### 5.4. Practical Deployment Considerations

MalariaNet at 85.3 KB in FP32 or 23.5 KB in INT8 is well within the deployment envelope of modern MCUs. Several practical considerations apply to real-world deployment.

Hardware cost. An STM32H7 development board costs approximately $12 (about 80 RMB for the MCU core module alone), and a medical-grade microscope camera module costs about $35. The total bill of materials therefore stays under $60. This is an order-of-magnitude reduction relative to a smartphone-based system (above $200) or a laptop-based system (above $500).

Power consumption. According to vendor data, the STM32H7 core draws on the order of 280 mW at full load. We did not measure device power, so this is a datasheet-level estimate rather than a validated figure. Battery operation is therefore plausible but not demonstrated here; full power, energy-per-inference and connectivity characterisation is left to future work.

Offline capability. Unlike cloud-based or smartphone-based solutions, MalariaNet on an MCU requires zero network connectivity, making it suitable for the most remote deployment scenarios.

### 5.5. Limitations

We acknowledge several limitations. The cross-dataset gap is large. Zero-shot BBBC041 accuracy is low under both protocols: 63.8% for the per-cell-trained protocol and 53.2±5.2% for the slide-disjoint protocol. No variant closes the *P. falciparum*-to-*P. vivax* and scanner shift without target-site adaptation. Species and life-cycle-stage labels are unavailable in NIH, so the multi-task burden head relies on auto-generated proxy labels. All in-domain training and evaluation use a single source dataset (NIH). Our same-species cross-laboratory test on MP-IDB (Section 4.7) is a first step and is encouraging, with the detector degrading gracefully rather than collapsing, but it covers a single additional laboratory and uses well-formed cell crops; a broader same-species validation across multiple laboratories, scanners and patient populations, ideally end-to-end on field captures and with a control that isolates our own cell-segmentation step from the laboratory shift, is still required before broad generalisation or deployment-readiness can be claimed. All in-domain data are *P. falciparum* single-cell patches. An end-to-end field device additionally needs cell detection and slide-level aggregation, which are not evaluated here. Deployment is validated for latency and memory on STM32H743 but not power. The compact-versus-large differential we noted (−0.25 to −0.47 pp pretrained vs. −0.86 to −1.45 pp from-scratch) is confounded with ImageNet pretraining. We addressed this with a controlled from-scratch experiment in which the three large baselines were retrained from scratch under both protocols with three seeds. Their leakage Δ values moved from −0.25, −0.39, and −0.47 to −0.75, −0.97, and −0.56 pp for ResNet-18, MobileNetV2, and EfficientNet-B0 respectively. The protection from pretraining is therefore architecture-dependent rather than universal, and we accordingly do not rely on the differential. The interference sweep uses programmatic perturbations as a proxy. The measured robustness advantage is therefore evidence under that controlled model, not a field guarantee. Most importantly, under leakage-free evaluation none of the investigated architectural elements provides a robust advantage, as established in Section 4.4 and Section 4.8. This paper’s positive contributions are methodological and practical, not a new architecture.

## 6. Conclusions

We set out to build a compact malaria detector for microcontrollers and found a more consequential problem: the field’s de facto benchmark leaks slide identity because its cells come from only about 200 patient slides yet are split per cell, mixing same-slide cells across training and test sets.

Under a leakage-free, slide-disjoint protocol, the same architecture loses roughly 1.5 percentage points of headline accuracy, but the more important effect is qualitative: every per-module ablation gain collapses into seed noise, and a variant that appeared to give a clear cross-site robustness advantage shrinks to within seed noise and changes sign. Thus, a protocol flaw can produce an internally consistent, publishable “innovation”. This benchmarking finding is this paper’s primary contribution and generalises to any medical-imaging benchmark where many patches share a patient or slide.

Rigorous evaluation leaves a single deployable baseline: a 21 K-parameter detector that reaches about 95.6% accuracy within the STM32H7 envelope, with a numerically faithful on-chip forward, that is the most interference-robust, microcontroller-deployable model in our leakage-free sweep. We claim no architectural superiority, since the ablations do not support it. The slide-disjoint split is fully reproducible from the public NIH file names. Because the system classifies pre-segmented single cells, a complete point-of-care device still needs image acquisition, detection, slide-level aggregation, and power and connectivity validation; the most useful next step is same-species, multi-site validation on real field images under leakage-free protocols.

## Figures and Tables

**Table 1:** Per-module parameter distribution of MalariaNet (total of 21,085 samples). MSME consumes 44.4% of the budget; the head consumes 34.1%, and the SCFE consumes only 3.4%, confirming that domain-specific stain priors are inexpensive while morphology encoding dominates the budget.

Component	Parameters	Percentage
Stem	1072	5.1%
SCFE (Stain stream)	721	3.4%
MSME (Morphology stream)	9376	44.4%
CDDG (Gate)	2682	12.7%
Head	7194	34.1%
BN & biases	40	0.2%
Total	21,085	100%

**Table 2:** Per-cell vs. slide-disjoint accuracy (3-seed mean), with all models retrained identically under both protocols. Δ is the per-cell minus slide-disjoint accuracy, i.e., the inflation attributable to slide leakage. The primary observation is that leakage inflates every (Δ<0 for all eight models). The apparent tendency for the compact from-scratch models to move more than the large ImageNet-pretrained backbones is confounded with the pretraining factor; a controlled from-scratch experiment (Section 5) shows this protective effect of pretraining is architecture-dependent, enlarging Δ for ResNet-18 and MobileNetV2 but not EfficientNet-B0, so we still do not treat the compact-versus-large differential as a primary result.

Model	Params	Size (KB)	Per-Cell Acc (%)	Slide-Disj. Acc (%)	Δ (pp)	MCU
ResNet-18	11.18 M	43,700	96.15	95.90±1.19	−0.25	No
MobileNetV2	2.23 M	8832	96.90	96.51±0.51	−0.39	No
EfficientNet-B0	4.01 M	15,832	97.22	96.75±0.60	−0.47	No
MobileNetV3-Small	1.52 M	5985	97.37	96.13±0.80	−1.24	No
ShuffleNetV2-x0.5	340 K	1375	97.58	96.56±0.36	−1.02	No
Tiny-MobileNetV2	89 K	375	96.69	95.83±0.71	−0.86	No
SingleStream	8 K	34	96.28	95.37±1.16	−0.91	Yes
MalariaNet (Ours)	21 K	85	97.06	95.61±1.02	−1.45	Yes

**Table 3:** Ablation under the leakage-free, slide-disjoint protocol (3-seed mean ± std). ΔAcc is full MalariaNet minus the variant. Under rigorous evaluation, every per-module difference is small relative to the ≈1 pp seed-to-seed standard deviation, and the SCFE and CDDG ablations are marginally negative; the individual module gains reported under the optimistic per-cell protocol (Section 4.1.2) do not survive leakage-free evaluation.

Configuration	Acc (%)	Sens (%)	Spec (%)	AUC	ΔAcc
SingleStream (no decoupling)	95.37±1.16	93.07	97.59	0.987	+0.24
w/o SCFE (no stain stream)	95.64±0.95	93.92	97.37	0.987	−0.03
w/o CDDG (simple concat)	95.69±1.05	94.06	97.33	0.989	−0.08
Uniform scale MSME	95.54±0.89	93.81	97.26	0.983	+0.07
Full MalariaNet	95.61±1.02	93.94	97.41	0.988	n/a

**Table 4:** Positive (PPV) and negative (NPV) predictive values of MalariaNet computed from the leakage-free, slide-disjoint operating point (sensitivity = 93.94%; specificity = 97.41%; 3-seed mean). PPV rises with prevalence; NPV stays ≥99% across the 0.5–10% endemic band.

Prevalence	PPV	NPV	Setting (Typical)
0.5%	15.4%	99.97%	Low-transmission elimination
1%	26.8%	99.94%	Mesoendemic, dry season
5%	65.6%	99.67%	Hyperendemic, baseline
10%	80.1%	99.31%	High endemic, sub-Saharan
20%	90.1%	98.5%	Peak-season epidemic
50%	97.3%	94.1%	Balanced split (reference)

**Table 5:** STM32H7 deployment feasibility. MalariaNet is the only entry that simultaneously satisfies the <200 KB Flash and <10 M FLOP MCU envelope while remaining MCU-deployable; the accuracy column reports the leakage-free, slide-disjoint accuracy (3-seed mean), consistent with the rest of the paper.

Model	Size (KB)	FLOPs (M)	<200 KB	Slide-Disj. Acc (%)
ResNet-18	43,700	595.4	No	95.90
MobileNetV2	8832	106.5	No	96.51
EfficientNet-B0	15,832	135.6	No	96.75
MobileNetV3-Small	5985	20.8	No	96.13
ShuffleNetV2-x0.5	1375	14.2	No	96.56
Tiny-MobileNetV2	375	11.0	No	95.83
SingleStream	34	3.3	Yes	95.37
MalariaNet	85	4.8	Yes	95.61

**Table 6:** STM32H743 on-device measured deployment metrics for the numerically faithful INT8-weight forward at the 128×128 evaluation resolution (DWT cycle counter, 10-iteration mean, and 400 MHz board clock). The on-chip classification matches the off-device reference to $1.8\times 10^{-4}$, so this is the real model rather than a workload proxy.

Metric	Measured Value
MCU	STM32H743IIT6 @ 400 MHz (board-limited; chip rated 480 MHz)
INT8 weights (Flash)	23.5 KB
Code size (Flash)	23.8 KB
Activation SRAM	489 KB
Latency (128×128, measured)	816.1 ms/326.4 M cycles (1.23 FPS)
Latency range (n=10)	816.0–816.3 ms (<0.3 ms)
On-chip kernel fidelity, max|C−ref|	$1.8\times 10^{-4}$

**Table 7:** Comparison with published methods on the NIH Malaria Cell Images dataset.

Method	Year/Venue	Params	Acc (%)	MCU-Deployable
Custom CNN [3]	2018/Transl. Res.	∼1 M	95.9	No
Fuhad et al. [4]	2020/Diagnostics	–	99.2	No
Islam et al. (ViT) [7]	2022/Sensors	∼86 M	95.0	No
Mujahid et al. [2]	2024/Sci. Reports	7.8 M	97.6	No
Chaudhry et al. [8]	2024/Neural Comp. App.	<400 K	97.1	No
UltraLightSqueezeNet-V3 [22]	2025/arXiv	∼120 K	96.6	Possible
MalariaNet (Ours), per-cell	–	21 K	97.06 ± 0.45	Yes
MalariaNet (Ours), slide-disjoint	–	21 K	95.61 ± 1.02	Yes

**Table 8:** Cross-dataset evaluation: trained on NIH and tested on BBBC041 (no fine-tuning).

Model	Acc (%)	Sens (%)	Spec (%)	AUC
SingleStream	48.44	97.55	8.30	0.539
Tiny-MobileNetV2	59.74	97.76	28.67	0.850
MalariaNet	63.79	96.33	37.20	0.830
MalariaNet w/o SCFE	74.03	88.42	62.27	0.873

**Table 9:** Same-species cross-laboratory zero-shot transfer. The deployed slide-disjoint MalariaNet, NIH-trained, evaluated with no fine-tuning on MP-IDB (*P. falciparum*, different laboratory) and, under the same slide-disjoint model and seeds, on BBBC041 (*P. vivax*, added species shift). Three-seed mean ± std. A same-species change of laboratory preserves a calibrated operating point; the further species shift collapses specificity.

External Set	Acc (%)	Sens (%)	Spec (%)	AUC
MP-IDB (*P. falciparum*, cross-lab)	79.5 ± 2.9	74.0 ± 8.3	82.7 ± 5.8	0.865 ± 0.029
BBBC041 (*P. vivax*, +species)	53.2 ± 5.2	96.4 ± 4.2	18.0 ± 12.8	0.768 ± 0.050

**Table 10:** The “cross-site robustness variant” is a training-time leakage artefact. Default MalariaNet vs. MalariaNet-P, 3-seed mean ± std, under per-cell training vs. leakage-free slide-disjoint training. The striking +7.0 pp BBBC041 advantage under per-cell training shrinks to +1.8 pp, within the ±5.5 pp seed noise and with inconsistent per-seed sign once training is leakage-free.

Training Protocol	Model	NIH Acc (%)	BBBC041 Acc (%)
Per-cell (leaky)	MalariaNet	97.06±0.45	63.79
	MalariaNet-P	96.65±0.14	70.82±1.45
Slide-disjoint	MalariaNet	95.61±1.02	53.23±5.23
	MalariaNet-P	95.69±0.82	55.04±5.69

**Table 11:** Leakage-free interference robustness (slide-disjoint, 3-seed). Models ranked by accuracy drop from S0 to S5 (smaller = more robust). MalariaNet is the second most robust of all eight CNNs, behind only the 530× larger, non-deployable ResNet-18 and, by a wide, seed-consistent margin, the most robust among MCU-deployable models.

Model	Params	S0 (%)	S5 (%)	Drop (pp)	MCU
ResNet-18	11.18 M	95.90	77.14	18.76±6.95	No
MalariaNet	21 K	95.61	73.78	21.84±6.10	Yes
Tiny-MobileNetV2	89 K	95.83	72.98	22.85±4.96	No
MobileNetV2	2.23 M	96.51	73.14	23.37±6.20	No
EfficientNet-B0	4.01 M	96.75	72.71	24.03±6.85	No
ShuffleNetV2-x0.5	340 K	96.56	68.33	28.23±6.95	No
SingleStream	8 K	95.37	66.79	28.59±8.98	Yes
MobileNetV3-Small	1.52 M	96.13	62.42	33.71±9.20	No

**Table 12:** Per-seed slide-disjoint test accuracy for the baseline 21 K detector and its knowledge-distillation student (α=0.7, T=4, EfficientNet-B0 teacher). The distillation change is non-positive at seed 42 and positive at seeds 123 and 2024; the +0.35 pp mean gain carries a paired 95% interval of [−0.73,+1.43] pp that includes zero, so it is a small variance-reducing mitigation rather than a statistically established improvement.

Seed	Baseline Acc (%)	KD Student Acc (%)	Δ (pp)	Test Cells
42	96.66	96.57	−0.09	3320
123	95.95	96.30	+0.35	3975
2024	94.24	95.02	+0.78	4719
Mean	95.61±1.02	95.96±0.68	+0.35	n/a

## Data Availability

The NIH Malaria Cell Images dataset is publicly available at https://lhncbc.nlm.nih.gov/publication/pub9932 (accessed on 15 April 2026), and the BBBC041 *P. vivax* dataset is available at https://bbbc.broadinstitute.org/BBBC041 (accessed on 15 April 2026). The leakage-free, slide-disjoint split is fully reproducible from the public NIH file names: it is a GroupShuffleSplit (70/15/15 by slide group, seeds 42/123/2024) on the leading C〈digits〉 slide identifier, exactly as specified in Section 4.1.2, so no additional artefact is required to reproduce the protocol or the leakage analysis. The source code, trained weights, INT8 checkpoints, and the STM32H743 Keil project are available from the corresponding author upon reasonable request.

## Abbreviations

The following abbreviations are used in this manuscript:

CNN	Convolutional Neural Network
MCU	Microcontroller Unit
POC	Point of Care
SCFE	Stain-Colour Feature Extractor
MSME	Multi-Scale Morphology Encoder
CDDG	Cross-Domain Diagnostic Gate
INT8	8-bit Integer Quantization
FP32	32-bit Floating Point
AUC	Area Under the Curve
ROC	Receiver Operating Characteristic
PPV	Positive Predictive Value
NPV	Negative Predictive Value
NIH	National Institutes of Health
BBBC	Broad Bioimage Benchmark Collection
RDT	Rapid Diagnostic Test
WHO	World Health Organization
